# Associations of Dietary Patterns, Daily Nutrient Intakes, and Food-Group Intakes with Hyperglycemia in Patients Undergoing Chemotherapy

**DOI:** 10.3390/nu18193249

**Published:** 2026-10-01

**Authors:** Kyung Hee Lim, Jiyeong Kim

**Affiliations:** College of Nursing, Keimyung University, Daegu 42601, Republic of Korea; jiyoung140326@gmail.com

**Keywords:** cancer, chemotherapy, hyperglycemia, dietary patterns, nutrient intake, food groups

## Abstract

**Background/Objectives:** Hyperglycemia can compromise anticancer treatment, leading to treatment delay or discontinuation. Identifying factors associated with hyperglycemia is therefore important. We aimed to examine associations between hyperglycemia and dietary patterns, food-group and nutrient intakes, and clinical and behavioral factors in patients undergoing chemotherapy. **Methods:** This cross-sectional survey assessed 176 patients with cancer undergoing chemotherapy at a university hospital in South Korea. Data were collected from April 2024 to March 2025 and analyzed using descriptive statistics, chi-square or Fisher’s exact tests, t-tests or Mann–Whitney U tests, and logistic regression. **Results:** Hyperglycemia was observed in 26.7% of participants. In adjusted analyses, the Korean side-dish dietary pattern (odds ratio [OR] = 0.30, 95% confidence interval [CI] 0.14–0.65, per 1 standard deviation increase in the pattern score), higher physical activity (highly active: OR = 0.06, 95% CI 0.01–0.31, active: OR = 0.13, 95% CI 0.04–0.40), and higher serum albumin levels (OR = 0.17, 95% CI 0.03–0.89) were associated with lower odds of hyperglycemia. Higher intakes of oils/fats, vegetables, eggs, and seaweed were associated with lower odds, whereas higher grain intake was associated with higher odds. All these associations remained significant after false discovery rate (FDR) correction. Because no individual nutrient remained significantly associated with hyperglycemia after FDR correction, nutrient-level findings were considered exploratory. **Conclusions:** Hyperglycemia was associated with the Korean side-dish dietary pattern, several food-group intakes, physical activity, and serum albumin. Given the observational design, these findings indicate associations rather than causal effects and warrant confirmation in prospective studies.

## 1. Introduction

Currently, the probability of developing cancer in the Korean population is 41.2%, up from 37.9% in 2021. In 2023, cancer prevalence was 1 in 19 people, compared with 1 in 25 in 2019 [1]. Cancer treatments fall into three categories: chemotherapy, surgery, and radiation therapy. Chemotherapy is administered to 60–70% of patients with cancer [2]. However, chemotherapy causes a range of adverse effects, including nausea, vomiting, hair loss, infection, anemia, skin changes, and hyperglycemia. Hyperglycemia has been reported in approximately 19.8% to 67% of patients receiving chemotherapy [3,4].

Certain anticancer agents can induce hyperglycemia by interfering with signaling pathways related to insulin and insulin-like growth factor, thereby impairing intracellular glucose transport and glycogen synthesis [5]. Glucocorticoids and other treatments may promote hepatic gluconeogenesis and reduce peripheral insulin sensitivity [4,6]. Representative drugs causing diabetes or hyperglycemia include cytotoxic anticancer agents such as 5-fluorouracil [6], platinum agents [7,8], and docetaxel [3,4], as well as glucocorticoids [4,6].

Hyperglycemia, an adverse effect of chemotherapy, can compromise anticancer treatment efficacy and worsen prognosis [3,9,10]. A hyperglycemic environment promotes tumor proliferation by supplying excess energy to tumor cells. Additionally, hyperinsulinemia resulting from insulin resistance has been reported to stimulate insulin receptors and insulin-like growth factor 1 receptors, thereby accelerating tumor cell proliferation and inhibiting apoptosis [10]. Patients who develop hyperglycemia after chemotherapy have a 9.7% higher risk of recurrence and a 2.6% lower 5-year survival rate than patients with normoglycemia [3]. Compared with patients with normoglycemia, those with hyperglycemia have more emergency department visits and unplanned hospitalizations, more frequent chemotherapy dose reductions, and a higher incidence of adverse events such as neutropenia, fever, and infection [9]. Therefore, preventing and managing hyperglycemia in patients undergoing chemotherapy are essential to minimize its adverse effects on the efficacy of anticancer treatment and prognosis. This requires identifying the factors that influence hyperglycemia and addressing them through nursing interventions.

Nonetheless, little research has examined associations between hyperglycemia and factors other than chemotherapeutic agents in patients with cancer undergoing chemotherapy. Research on dietary factors in this population remains limited in Korea and internationally, particularly regarding the relationship between dietary patterns and hyperglycemia. The dietary-pattern approach rests on the premise that nutrients and food groups interact in complex ways within the overall diet rather than being consumed in isolation. Looking beyond individual nutrients, this approach may capture the multidimensional nature of diet and its complex metabolic associations [11]. Therefore, we aimed to identify dietary patterns among patients undergoing chemotherapy and to examine the associations of these patterns, daily nutrient intake, and food-group intake with hyperglycemia. The findings were intended to inform the development of programs to help prevent and manage hyperglycemia.

## 2. Materials and Methods

### 2.1. Study Design

This descriptive survey examined associations between hyperglycemia and dietary patterns, daily nutrient intake, and food-group intake in patients with cancer undergoing chemotherapy. The study was conducted in accordance with the Declaration of Helsinki and was approved by the Institutional Review Board (IRB No. 2024-02-016-001) of K University D Hospital in D Metropolitan City. The Strengthening the Reporting of Observational Studies in Epidemiology—Nutritional Epidemiology (STROBE-nut) guidelines were also followed in the study design and reporting. Developed for nutritional epidemiology and dietary assessment research, STROBE-nut is an extension of the STROBE Statement.

### 2.2. Study Participants

Between April 2024 and March 2025, patients undergoing chemotherapy at K University D Hospital in D Metropolitan City were included. Previous studies evaluating chemotherapy-related hyperglycemia across repeated treatment cycles [3,4] were used to inform inclusion criteria.

Eligibility was based on four criteria: (1) having cancer, not having been diagnosed with diabetes mellitus before initiating chemotherapy, and having completed three or more chemotherapy cycles; (2) receiving a chemotherapy regimen containing 5-fluorouracil, cisplatin, carboplatin, or docetaxel; (3) receiving 8–12 mg of dexamethasone as an antiemetic; and (4) being aged 18 years or older, having sufficient communication skills to complete the questionnaire, and providing voluntary informed consent to participate. Patients were excluded for any of the following reasons: (1) receipt of total parenteral nutrition during the data collection period; (2) terminal cancer or previous pancreatic surgery; (3) concurrent steroid therapy for diseases other than cancer; or (4) failure to complete the questionnaire.

**Figure 1:**
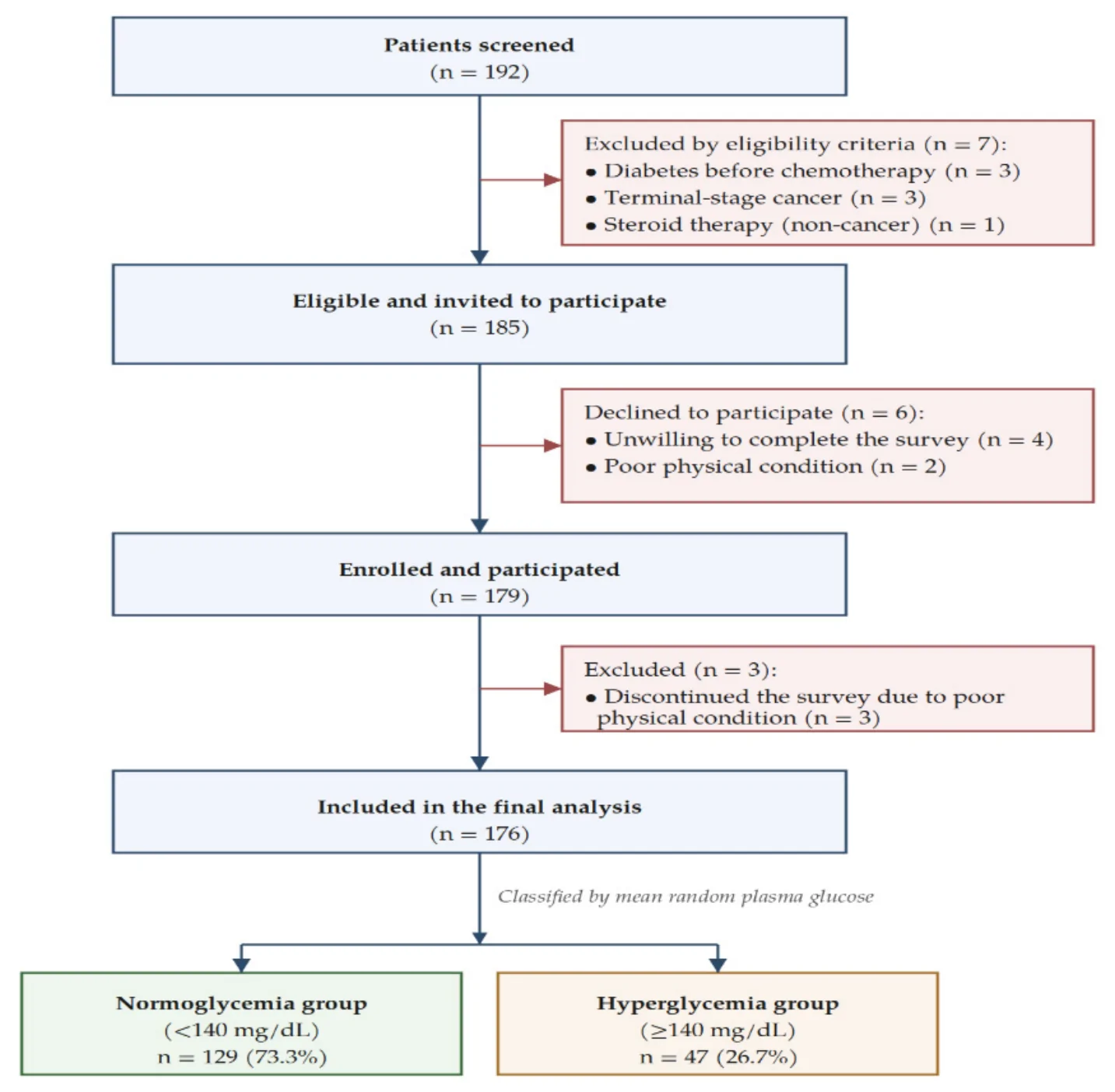
Participant flow diagram.

The required sample size was calculated using G*Power 3.1.9.4 (Heinrich-Heine-Universität Düsseldorf, Düsseldorf, Germany) and assumptions from a previous study [3]: a significance level of 0.05, statistical power of 80%, and an odds ratio (OR) of 1.8. For logistic regression analysis, the minimum sample size was 152 participants. To allow for an anticipated attrition rate of 15%, the target sample size was 179 participants. Of the 192 patients screened, seven were excluded based on the eligibility criteria: three had diabetes diagnosed before chemotherapy, three had terminal-stage cancer, and one was receiving steroid therapy for a non-cancer disease. Of the 185 eligible patients invited to participate, six declined: four were unwilling to complete the survey, and two could not respond because of poor physical condition. The remaining 179 patients enrolled. During data collection, three participants discontinued the survey because of poor physical condition and were excluded; thus, 176 participants were included in the final analysis (Figure 1). No participants were excluded because of biologically implausible dietary intake values.

### 2.3. Research Instruments

Data on six general characteristics (sex, age, marital status, educational level, alcohol consumption, and smoking status) and two exercise-related factors (weekly exercise frequency and exercise duration per session) were collected using a structured questionnaire. Dietary patterns, daily nutrient intake, and food-group intake were assessed using a 112-item food frequency questionnaire (FFQ); dietary supplement intake was not assessed. From electronic health records (EHRs), disease- and treatment-related factors (diagnosis, clinical stage, body mass index, comorbidities, treatment cycle, and concomitant medications), serum albumin levels, and random plasma glucose levels were obtained.

#### 2.3.1. Random Plasma Glucose

The primary objective was to identify hyperglycemia occurring during systemic anticancer treatment rather than to diagnose pre-existing diabetes; therefore, random plasma glucose (RPG) was used instead of fasting plasma glucose (FPG) or glycated hemoglobin (HbA1c). Because treatment-related elevations in blood glucose may be more pronounced outside the fasting state, FPG may not adequately capture treatment-related hyperglycemia, particularly in patients receiving glucocorticoids [4]. Unlike HbA1c, which reflects longer-term glycemic exposure and may therefore fail to capture acute or transient increases in blood glucose during chemotherapy [12], RPG reflects current glycemic status without requiring fasting. Cancer-specific guidance recommends that HbA1c and plasma glucose be assessed before systemic anticancer and/or glucocorticoid therapy and that RPG be monitored at each treatment visit [13].

Following a previous study [3], random plasma glucose was calculated, regardless of fasting status, as the mean of the routine pre-chemotherapy values from the cycle at the time of the survey and the immediately preceding cycle. Blood samples were collected approximately 3 h before chemotherapy and 2.5 h before dexamethasone premedication to minimize their potential acute effects on blood glucose levels and to maintain comparable measurement conditions across treatment cycles. Meal timing relative to blood sampling was not systematically recorded. Because samples were collected under routine pre-chemotherapy conditions rather than under standardized fasting conditions, variation in the time since the last meal may have contributed to measurement variability. The two pre-treatment glucose measurements were averaged to reduce the influence of short-term within-person variability. Hyperglycemia was defined as a mean random plasma glucose level ≥ 140 mg/dL, consistent with previous research on chemotherapy-related hyperglycemia [3].

#### 2.3.2. Weekly Exercise Volume

A modified version of the tool developed by Hong (2006) [14] was used to assess weekly exercise volume. The original tool was designed to assess the type and amount of exercise in patients with diabetes mellitus. Using the energy expenditure chart for exercise and physical workload by McArdle et al. (2004) [15], total weekly energy expenditure was calculated from each patient’s body weight, exercise type, and exercise duration. This value represented weekly exercise volume. For analysis, participants were classified into three activity groups based on a prior study [16]: “inactive” (less than 1000 kcal/week), “active” (1000 to less than 2000 kcal/week), and “highly active” (2000 kcal/week or more). Weekly exercise volume was calculated using the following formula:

{Weekly exercise volume (kcal)} = {Calories burned by activity and body weight} × {Exercise duration per session (min)} × {Weekly exercise frequency}

#### 2.3.3. Dietary Patterns

Dietary intake was assessed using the semi-quantitative food frequency questionnaire used in the 7th Korea National Health and Nutrition Examination Survey (KNHANES VII). The instrument’s reproducibility and validity have been reported previously [17]; it assesses the average frequency and portion size of food intake over the preceding 12 months. For each food item, consumption frequency was recorded on a nine-category scale (ranging from “almost never” to “three or more times per day”), and usual portion size was recorded on a three-level scale (0.5, 1, or 1.5 servings relative to a standard reference portion). For seasonal foods, such as fruits, intake was assessed by season and converted to an annual average. Daily intake of each food was estimated by multiplying the reported frequency by the portion size and the standard reference amount. Food items were then aggregated into food groups for analysis. Each participant’s daily nutrient and food-group intakes were calculated using CAN-Pro 4.0 (2011), nutritional assessment software developed by the Korean Nutrition Society (Seoul, Republic of Korea). For quality control, records with biologically implausible total energy intakes were reviewed, and data entry was double-checked. Dietary supplement intake was not assessed. The KNHANES FFQ was developed and validated in the general Korean adult population [17], and separate FFQs have also been developed and validated for Korean patients with cancer and breast cancer survivors [18,19]. Factor analysis (principal component analysis) was subsequently applied to food-group intakes to identify distinct dietary patterns.

### 2.4. Data Collection Procedures

A survey was administered in a private counseling room at the hospital’s cancer center after approval was obtained from the Institutional Review Board (IRB No. 2024-02-016-001) of K University D Hospital in D Metropolitan City. Before administering the survey, the study’s purpose, procedures, and time required were explained to participants, who were assured that their questionnaire and EHR data would remain confidential and would be used only for the stated research purposes. Written informed consent was obtained from all participants. During the face-to-face survey, each item was read aloud by the researcher, who recorded participants’ responses. The mean completion time was approximately 30 min. During the same period, participants’ disease-related characteristics, serum albumin levels, and random plasma glucose levels were retrieved from their EHRs by the researchers.

### 2.5. Statistical Analysis

Data were analyzed using IBM SPSS Statistics for Windows, version 30.0 (IBM Corp., Armonk, NY, USA). General and disease-related categorical characteristics were compared between participants with and without hyperglycemia using the chi-square test or Fisher’s exact test, as appropriate. Normality of continuous variables was assessed using the Shapiro–Wilk test. Normally and non-normally distributed variables were compared using the independent t-test and Mann–Whitney U test, respectively. Dietary patterns were identified by factor analysis using principal component analysis (PCA) with varimax orthogonal rotation. Absolute (unadjusted) daily food-group intakes were used because factor analysis characterizes the correlation structure among food groups rather than the intake of any single component. After principal component extraction and varimax rotation, individual factor scores were computed using the regression method. The resulting scores were standardized and mutually orthogonal (mean 0, standard deviation [SD] 1). These scores were entered into the logistic regression models; the odds ratios for dietary patterns therefore represent the change in odds per 1 SD increase in the pattern score. For each factor, food groups with absolute loadings of 0.40 or higher were identified in the rotated component matrix. These dietary patterns were named with reference to the Korean National Standard Food Composition Table [20]. Daily nutrient and food-group intakes were energy-adjusted using the residual method and expressed per 1 SD. Multiple comparisons for nutrients and food groups were controlled using the Benjamini–Hochberg false discovery rate (FDR) procedure. Descriptive statistics (mean ± SD and median [IQR]) for the continuous dietary exposures are provided in Supplementary Table S1. For the food groups significantly associated with hyperglycemia, linearity in the logit was examined by categorizing the energy-adjusted intakes into quartiles and testing for a linear trend; a significant monotonic trend was observed for all of these exposures (all *p* for trend < 0.05), supporting the assumption of an approximately linear association on the logit scale. Factors associated with hyperglycemia were evaluated using logistic regression, and statistical significance was set at *p* < 0.05.

Covariates were prespecified based on prior knowledge and biological plausibility rather than selected solely according to univariate statistical significance. The primary models for dietary patterns, food groups, and nutrients were adjusted for age, educational level, physical activity level, and serum albumin. The sensitivity model was additionally adjusted for sex, body mass index (BMI), cancer diagnosis (gastrointestinal vs. other), disease stage (stage IV vs. stages I–III), and number of chemotherapy cycles. To address a broader range of potential confounders, antihypertensive medication use, immunotherapy, and cumulative dexamethasone dose were added to an extended sensitivity model. Because hypertension and antihypertensive medication use were collinear, they were not entered simultaneously. Because the fully adjusted extended model had a low events-per-variable ratio, it was treated as exploratory, and Firth-penalized logistic regression was also fitted to reduce small-sample and sparse-data bias and assess the robustness of the estimates. The primary model was evaluated for multicollinearity using variance inflation factors, and model fit was assessed using the Hosmer–Lemeshow goodness-of-fit test and the Cox & Snell and Nagelkerke pseudo-R^2^ statistics.

## 3. Results

### 3.1. General and Disease-Related Characteristics

Table 1 summarizes the general and disease-related characteristics of participants with and without hyperglycemia. Of the participants, 47 (26.7%) were classified as having hyperglycemia, and 129 (73.3%) as having normoglycemia. The groups differed significantly in age (χ^2^ = 18.70, *p* = 0.001) and educational attainment (χ^2^ = 20.01, *p* = 0.001). In the hyperglycemia group, the largest age and educational categories were 70 years or older and elementary school or less, respectively (59.6% and 46.8%). In the normoglycemia group, the largest age category was < 60 years (43.4%), and high school graduates formed the largest educational category (41.9%).

Among disease-related characteristics, the groups differed significantly in comorbidity status (χ^2^ = 5.02, *p* = 0.028), hypertension (χ^2^ = 6.64, *p* = 0.017), cardiovascular disease (Fisher’s exact test, *p* = 0.012), antihypertensive medication use (χ^2^ = 6.64, *p* = 0.017), and mean serum albumin level (t = 5.21, *p* < 0.001). In the hyperglycemia group, 29 (61.7%) participants had comorbidities, whereas 18 (38.3%) did not. Conversely, most participants in the normoglycemia group had no comorbidities (74 participants, 57.4%). Hypertension was more common in the hyperglycemia group (46.8%) than in the normoglycemia group (26.4%). Other cardiovascular diseases were more prevalent in the hyperglycemia group than in the normoglycemia group (12.8% and 2.3%, respectively). Antihypertensive medication use was also more common in the hyperglycemia group (46.8%) than in the normoglycemia group (26.4%). The mean serum albumin level was lower in the hyperglycemia group (3.87 g/dL) than in the normoglycemia group (4.19 g/dL). The cumulative dexamethasone dose did not differ between the groups (median, 80 mg in both, Mann–Whitney U test, *p* = 0.959). Hypertension and antihypertensive medication use had identical frequencies and were effectively collinear because all patients with hypertension were receiving antihypertensive medication. To avoid redundancy, only one of these variables was retained as a candidate predictor in the regression models.

### 3.2. Weekly Exercise Volume

Table 2 presents participants’ weekly exercise measures. Because the Shapiro–Wilk test indicated that these measures were non-normally distributed (all *p* < 0.001), they were compared between groups using the Mann–Whitney U test and summarized as medians (interquartile range [IQR]). All exercise measures differed significantly between the groups.

Compared with the hyperglycemia group, the normoglycemia group had a higher median weekly exercise frequency and longer median exercise duration per session: 6 times/week [IQR 3–7] versus 0 [IQR 0–5] (Z = 5.42, *p* < 0.001) and 60 min [IQR 40–90] versus 0 [IQR 0–30] (Z = 6.71, *p* < 0.001), respectively. Weekly exercise volume was also greater in the normoglycemia group: the median was 1680 kcal/week [IQR 987–2380], compared with 0 [IQR 0–723] in the hyperglycemia group (Z = 7.29, *p* < 0.001).

### 3.3. Classification of Dietary Patterns

PCA with varimax orthogonal rotation was applied to participants’ daily intakes of 15 food groups to identify dietary patterns. Table 3 summarizes the factor-analysis results. The Kaiser–Meyer–Olkin measure of sampling adequacy was 0.794, indicating that the data were suitable for factor analysis. Bartlett’s test of sphericity was also significant (χ^2^ = 951.57, *df* = 105, *p* < 0.001), suggesting that correlations among the variables were sufficient to support factor analysis.

Kaiser’s criterion yielded five factors with eigenvalues of 1.0 or higher. Together, they explained 64.5% of the variance. Factor 1 explained the largest proportion of variance (22.39%), followed by Factors 2, 3, 4, and 5, which explained 14.24%, 10.33%, 9.10%, and 8.46%, respectively. Because several food groups loaded on more than one factor, each was assigned to the factor with the highest absolute loading.

The five patterns were named based on food groups with absolute loadings ≥ 0.40 in the rotated component matrix and with reference to the Korean National Standard Food Composition Table. Factor 1 was the “Korean side-dish pattern,” comprising oils/fats, seaweed, mushrooms, vegetables, condiments/seasonings, meats, and eggs; Factor 2 was the “grain-sugar and fish pattern,” comprising grains, sugars, and fish/shellfish; and Factor 3 was the “dairy-fruit pattern,” comprising milk/dairy products and fruits. Factor 4 was the “nuts/seeds-tubers pattern,” comprising nuts/seeds and potatoes/tubers; Factor 5 was the “legume pattern,” comprising legumes.

### 3.4. Factors Associated with Hyperglycemia

Logistic regression was used to examine factors associated with hyperglycemia among patients undergoing chemotherapy. Covariates were selected based on prior knowledge and biological plausibility rather than univariate significance alone. The dietary-pattern, food-group, and nutrient models were adjusted for the following clinically motivated covariates: age, education, physical activity level, and serum albumin (Table 4). Food-group and nutrient intakes were energy-adjusted using the residual method and expressed per 1 SD; dietary-pattern scores were derived from absolute food-group intakes and standardized separately. The Benjamini–Hochberg FDR correction was applied to the food-group and nutrient analyses.

We also performed an extended sensitivity analysis to account for a broader range of potential confounders. The model simultaneously included sex, BMI, cancer diagnosis, disease stage, number of chemotherapy cycles, other medications (antihypertensive medication and immunotherapy), and cumulative dexamethasone dose (Supplementary Table S2). The core associations involving the Korean side-dish dietary pattern, physical activity, and serum albumin remained robust. Cumulative dexamethasone was not significantly associated with hyperglycemia (OR = 1.44, 95% confidence interval [CI]: 0.77–2.68, *p* = 0.255). However, the fully adjusted model is presented as a sensitivity analysis rather than as the primary model because it had a low events-per-variable ratio (2.35) and yielded unstable estimates for some variables (e.g., immunotherapy OR = 68.50, 95% CI: 1.34–3514.09).

Because the events-per-variable ratio was low, we also fitted the fully adjusted model with Firth-penalized logistic regression to further assess the robustness of the estimates (Supplementary Table S2). Firth penalization markedly stabilized the estimates for sparse variables. For example, the standard OR for immunotherapy was 68.50 (95% CI, 1.34–3514.09), compared with a Firth OR of 21.88 (95% CI, 0.95–506.04). Both methods yielded significant results for the core associations—the Korean side-dish pattern, physical activity, and serum albumin—whereas cumulative dexamethasone remained non-significant (Firth OR = 1.28, *p* = 0.356).

Diagnostic checks confirmed that the primary model was adequate, with Cox & Snell and Nagelkerke pseudo-R^2^ values of 0.395 and 0.575, respectively. Multicollinearity was acceptable, with a maximum variance inflation factor of 2.46. The Hosmer–Lemeshow test indicated a good fit (χ^2^ = 1.74, *p* = 0.988).

The primary model included the dietary pattern and the prespecified clinical and behavioral covariates (Table 4). The Korean side-dish dietary pattern (Factor 1) was associated with lower odds of hyperglycemia per 1 SD increase in the pattern score (OR = 0.30, 95% CI: 0.14–0.65, *p* = 0.002). Compared with the inactive group, the active and highly active groups had lower odds of hyperglycemia (ORs of 0.13; 95% CI: 0.04–0.40, and 0.06; 95% CI: 0.01–0.31, respectively). Each 1 g/dL increase in serum albumin was associated with lower odds of hyperglycemia (OR = 0.17, 95% CI: 0.03–0.89). In the sensitivity model, these associations remained robust after additional adjustment for sex, BMI, cancer diagnosis, disease stage, and chemotherapy cycles (Table 4). Gastrointestinal cancer was significant in the sensitivity model (Table 4), and female sex was also significantly associated with hyperglycemia in the extended model (Supplementary Table S2).

In the food-group analysis, intakes were adjusted for energy and expressed per 1 SD. The Benjamini–Hochberg FDR procedure was used to control for multiple testing (Table 5). After FDR correction, five food groups were associated with hyperglycemia. Higher intakes of oils/fats (OR = 0.31, 95% CI: 0.16–0.57, *q* = 0.002), vegetables (OR = 0.30, 95% CI: 0.15–0.57, *q* = 0.002), eggs (OR = 0.34, 95% CI: 0.16–0.71, *q* = 0.015), and seaweed (OR = 0.32, 95% CI: 0.14–0.75, *q* = 0.020) were associated with lower odds of hyperglycemia. Higher grain intake, however, was associated with higher odds (OR = 1.90, 95% CI: 1.19–3.02, *q* = 0.020).

In the exploratory nutrient analysis, none of the individual nutrients remained significantly associated with hyperglycemia after FDR correction (all *q* > 0.05; Table 6). Before correction, higher intakes of several nutrients, including vitamin E, cholesterol, folate, and dietary fiber (all per 1 SD), were associated with nominally lower odds of hyperglycemia.

Neither age nor educational level was significantly associated with hyperglycemia in the adjusted models. Table 4 and Supplementary Table S2 provide detailed estimates for all variables in the primary and sensitivity models, including the extended analysis incorporating cumulative dexamethasone and other medications.

## 4. Discussion

This study examined the associations of dietary patterns, daily nutrient intake, and food-group intake with hyperglycemia in patients undergoing chemotherapy and aimed to inform interventions to prevent and manage hyperglycemia in patients with cancer. Among the 176 participants, 47 (26.7%) had hyperglycemia, a proportion within the 19.8–67% range reported in previous studies [3,4]. Hyperglycemia may partly reflect cancer- and treatment-related metabolic alterations, including impaired insulin secretion and increased insulin resistance [6,21]. By increasing the likelihood of cancer recurrence, mortality, and treatment-related adverse events, hyperglycemia contributes substantially to poor outcomes of cancer therapy [3,9]. To develop nursing interventions to prevent and manage hyperglycemia in patients with cancer, it is therefore important to identify modifiable factors associated with the condition. By identifying dietary and other modifiable factors associated with hyperglycemia during chemotherapy, this study contributes to this evidence.

A 1 SD increase in the Korean side-dish dietary pattern score was associated with 70% lower odds of hyperglycemia. Characteristic of the traditional Korean diet, this pattern comprises oils/fats, seaweed, mushrooms, vegetables, condiments/seasonings, meats, and eggs; its side dishes (banchan) are typically prepared with vegetable oils, such as perilla or sesame oil, and fermented condiments. Several components of this pattern may contribute to postprandial glucose stabilization through multiple pathways. Abundant in vegetables, seaweed, and mushrooms, soluble dietary fiber can slow gastric emptying and reduce glucose absorption [22], while vegetable-derived unsaturated fats, when consumed alongside carbohydrates, may attenuate the postprandial glucose rise [23,24].

These mechanisms may partly explain the association with lower odds of hyperglycemia observed in this study. However, a causal relationship between the Korean side-dish pattern and hyperglycemia cannot be established given the cross-sectional study design. Reverse causation, whereby patients with better health status were better able to maintain this dietary pattern, also cannot be ruled out. Experimental and observational studies have reported potentially favorable metabolic associations with fermented soybean products [25,26]. Nonetheless, fermented condiments are also a major dietary source of sodium, so the role of this food group in the overall pattern warrants cautious interpretation. Prospective or interventional studies are needed to clarify the relationship between this dietary pattern and glycemic control in patients undergoing chemotherapy.

Higher intakes of vegetables, oils/fats, seaweed, and eggs were associated with lower odds of hyperglycemia, whereas higher grain intake was associated with higher odds. These food-group associations remained significant after FDR correction. Previous evidence linking vegetables and soluble dietary fiber to improved glycemic regulation and a lower risk of prediabetes is consistent with the inverse association observed for vegetable intake [22,27]. Future studies are needed to confirm the observed associations for oils/fats, seaweed, and eggs.

Higher grain intake was associated with higher odds of hyperglycemia. However, this association should be interpreted with caution because this study did not distinguish among grain types. Previous studies indicate that the association with hyperglycemia differs by grain type. White rice and refined grains have been associated with an increased risk of diabetes, whereas whole-grain intake has been associated with a lower incidence of type 2 diabetes [28,29,30]. CAN-Pro 4.0 classified white rice, whole grains, noodles, bread, rice cakes, and cereals into a single category in this study, limiting our ability to examine the association between individual grain types and hyperglycemia. Future studies should therefore distinguish grain subtypes. Previous findings suggest that replacing refined grains with whole grains may be more beneficial than indiscriminately reducing total grain intake.

Higher daily intakes of vitamin E, cholesterol, folate, and dietary fiber were associated with lower odds of hyperglycemia. However, no individual nutrient retained a significant association with hyperglycemia after multiple-comparison correction using the Benjamini–Hochberg FDR method (all *q* > 0.05). The pre-correction associations should therefore be interpreted as exploratory findings requiring further investigation rather than as confirmatory results. The absence of any FDR-significant nutrient association suggests that glycemic status may be associated with overall dietary composition and with disease- and treatment-related metabolic changes, rather than with the intake of any single nutrient.

Several methodological factors should also be considered when interpreting the absence of significant associations with individual nutrients. Because nutrients are highly intercorrelated and often co-occur in the same foods, it is difficult to isolate the independent contribution of any single nutrient. In addition, intake estimates from a food frequency questionnaire are subject to recall bias and measurement error. The limited number of hyperglycemia events (*n* = 47) may have provided insufficient statistical power to detect small effects. Moreover, patients with cancer undergoing chemotherapy may experience increased inflammation, oxidative stress, insulin resistance, and muscle protein breakdown. These metabolic changes are known to affect both blood glucose homeostasis and amino-acid utilization and metabolism [31]. Therefore, it would be inappropriate to draw clinical conclusions recommending or restricting the intake of specific nutrients based on the present findings. Future prospective studies with larger samples are needed to include dietary assessments alongside concurrent measurements of blood nutrient levels, amino-acid concentrations, inflammatory markers, and body composition, as well as assessments of insulin resistance and sarcopenia.

Beyond dietary factors, weekly exercise volume and serum albumin were also associated with hyperglycemia in this study. Greater weekly exercise volume was associated with lower odds of hyperglycemia; the active and highly active groups had markedly lower odds than the inactive group. Previous studies have reported that exercise can improve insulin sensitivity and skeletal-muscle glucose utilization [32]. Exercise interventions during chemotherapy have also been associated with improvements in fasting glucose and HbA1c [33]. Diabetes clinical guidelines also recommend regular moderate-intensity physical activity for glycemic control [34,35]. However, the cross-sectional design of this study means that the observed association cannot be interpreted as an effect of exercise itself. Patients in poorer clinical condition—owing to reduced performance status, cancer-related fatigue, cachexia, or treatment toxicity—may be less able to exercise and may also be more prone to metabolic dysregulation; reverse causation is therefore possible. Residual confounding by these factors may also partly account for the observed association. Nevertheless, combining aerobic exercise with appropriate resistance exercise may help preserve muscle mass in patients with cancer. Individualized exercise support tailored to each patient’s clinical condition and treatment situation is therefore warranted. The association between exercise and hyperglycemia also requires confirmation in prospective interventional studies.

Among disease-related factors, higher serum albumin levels were associated with lower odds of hyperglycemia. This finding is consistent with population-based evidence linking serum albumin to incident diabetes risk [36] and with observations that hypoalbuminemia is more pronounced among patients with both cancer and type 2 diabetes [37]. To date, a direct causal relationship between albumin and hyperglycemia has not been clearly established. Nonetheless, low albumin levels may be accompanied by increased oxidative stress and inflammatory activity, which can affect insulin resistance [38,39]. Low albumin levels are also associated with reduced muscle mass [40]. Decreased skeletal-muscle mass may impair glucose utilization and adversely affect glycemic control [32]. In light of these findings and our results, serum albumin is better interpreted as a clinical marker reflecting overall nutritional status, inflammation, and muscle loss rather than a causal determinant of glycemic control. Serum albumin may help identify patients who need closer nutritional and metabolic assessment, but prospective studies are needed to confirm the observed association.

This study has some limitations. First, the study was conducted at a single center, limiting the generalizability of the findings. Second, the cross-sectional design precludes conclusions about a causal relationship between dietary factors and hyperglycemia. Moreover, residual confounding by cancer characteristics and treatment-related factors (e.g., performance status, disease stage, and chemotherapy regimen) cannot be ruled out. Third, dietary assessment had limitations in several respects. Because dietary intake was estimated using a self-reported questionnaire, it may be affected by recall bias and underreporting. Although the food frequency questionnaire was validated in Korean adults and has been used in previous studies of patients with cancer, its validity in patients undergoing chemotherapy has not been separately established. In addition, the quartile-based trend analysis assessed monotonicity only for the five FDR-significant food groups and did not formally exclude nonlinear associations between continuous dietary exposures and the log odds of hyperglycemia. Treatment-related symptoms such as appetite loss, nausea, and taste alterations may have introduced additional measurement error into the intake estimates. Additionally, the food-composition database did not distinguish refined grains from whole grains, precluding evaluation of differences by grain type. Dietary supplement intake was not assessed and thus was not reflected in the nutrient estimates. Nutrient estimates, including those for fluorine and amino acids, were based on a food-composition table rather than objective biomarkers and may not reflect circulating concentrations. Fourth, fasting glucose and HbA1c were not consistently available for all participants, precluding the use of these measures to define or analyze hyperglycemia. Because meal timing relative to blood sampling was not systematically recorded, variation in the interval since the last meal may have increased measurement variability and contributed to outcome misclassification. Fifth, with only 47 hyperglycemia events, the study was underpowered to detect small effects or obtain stable estimates from models with many predictors. Although the parsimonious primary model showed acceptable diagnostics, some estimates had wide confidence intervals, reflecting limited statistical power. The fully adjusted analyses should therefore be regarded as exploratory.

## 5. Conclusions

In this cross-sectional study, hyperglycemia was identified in 26.7% of patients undergoing chemotherapy and was associated with the Korean side-dish dietary pattern, food-group intakes, physical activity, and serum albumin. In particular, lower odds of hyperglycemia were associated with the dietary pattern characterized by traditional Korean side-dish food groups, higher levels of physical activity, and higher serum albumin. After correction for multiple testing, higher intakes of oils/fats, vegetables, eggs, and seaweed were associated with lower odds of hyperglycemia, whereas higher grain intake was associated with higher odds. Across the dietary-pattern and food-group analyses, vegetable, seaweed, and oils/fats intakes were consistently associated with lower odds of hyperglycemia. Although some individual nutrients showed nominal associations with hyperglycemia, none remained significant after FDR correction. These findings were therefore regarded as exploratory.

These findings suggest that nutritional counseling for patients undergoing chemotherapy should consider overall dietary composition, food-group balance, physical activity, and nutritional status together rather than emphasize any single nutrient or food in isolation. However, because this is an observational study, the findings should not be interpreted as a basis for directly prescribing specific dietary intake or exercise. Rather, they may serve as preliminary evidence for developing individualized strategies to manage hyperglycemia that account for each patient’s clinical condition and treatment situation.

## Figures and Tables

**Table 1:** General and disease-related characteristics of participants with and without hyperglycemia.

Characteristic		Category		Normoglycemia (*n* = 129)	Hyperglycemia (*n* = 47)	χ^2^, *t* or *U*	*p*
				*n* (%) Mean ± SD	*n* (%) Mean ± SD		
General	Sex	Male		71 (55.0)	31 (66.0)	1.69	0.229
Characteristics		Female		58 (45.0)	16 (34.0)		
	Age	<60 years		56 (43.4)	6 (12.7)	18.70	0.001 *
		60–69 years		37 (28.7)	13 (27.7)		
		≥70 years		36 (27.9)	28 (59.6)		
	Marital	Married		101 (78.3)	40 (85.1)	1.00	0.396
	status	Other		28 (21.7)	7 (14.9)		
	Educational	Elementary School or lower		29 (22.5)	22 (46.8)	20.01	0.001 *
	attainment	Middle school		16 (12.4)	12 (25.5)		
		High school		54 (41.9)	10 (21.3)		
		College/University or higher		30 (23.2)	3 (6.4)		
	Alcohol	No		125 (96.9)	44 (93.6)	-	0.386 ^a^
	consumption	Yes		4 (3.1)	3 (6.4)		
	Smoking	No		126 (97.7)	45 (95.7)	-	0.611 ^a^
	status	Yes		3 (2.3)	2 (4.3)		
Disease	Diagnosis	Gastrointestinal cancer		71 (55.0)	29 (61.6)	-	0.490 ^a^
related		Breast cancer		11 (8.5)	2 (4.3)		
Characteristics		Lung cancer		14 (10.9)	6 (12.8)		
		Genitourinary cancer		27 (20.9)	10 (21.3)		
		Other		6 (4.7)	0 (0.0)		
	Disease	Stage I		3 (2.4)	0 (0.0)	-	0.248 ^a^
	stage	Stage II		15 (11.7)	6 (12.8)		
		Stage III		31 (24.2)	6 (12.8)		
		Stage IV		79 (61.7)	35 (74.4)		
	Body Mass	Underweight (<18.5 kg/m^2^)		7 (5.5)	3 (6.4)	-	0.519 ^a^
	Index	Normal (18.5–22.9 kg/m^2^)		55 (42.6)	15 (31.9)		
		Overweight (23.0–24.9 kg/m^2^)		31 (24.0)	11 (23.4)		
		Obesity (≥25.0 kg/m^2^)		36 (27.9)	18 (38.3)		
	Comorbidities	None		74 (57.4)	18 (38.3)	5.02	0.028 *
		Present		55 (42.6)	29 (61.7)		
	Hypertension	None		95 (73.6)	25 (53.2)	6.64	0.017 *
		Present		34 (26.4)	22 (46.8)		
	Cardiovascular disease	None		126 (97.7)	41 (87.2)	-	0.012 ^a^ *
		Present		3 (2.3)	6 (12.8)		
	Treatment	Cycles 3–4		28 (21.7)	12 (25.5)	2.54	0.469
	cycle	Cycles 5–6		37 (28.7)	8 (17.0)		
		Cycles 7–12		36 (27.9)	16 (34.0)		
		Cycle 13 or higher		28 (21.7)	11 (23.5)		
	Other	Antihypertensive	No	95 (73.6)	25 (53.2)	6.64	0.017 *
	medications		Yes	34 (26.4)	22 (46.8)		
		Appetite	No	119 (92.2)	38 (80.9)	4.65	0.060
		Stimulants	Yes	10 (7.8)	9 (19.1)		
		Cumulative Dexamethasone Dose (mg)		80.0 (45.0–154.0)	80.0 (49.0–141.0)	1948	0.959 ^b^
	Blood test	Mean Serum Albumin (g/dL) ^†^		4.19 ± 0.38	3.87 ± 0.32	5.21	<0.001 *

^a^ Fisher’s exact test was used; all other categorical comparisons used the chi-square test. ^b^ The Mann–Whitney U test was used. The cumulative dexamethasone dose is summarized as the median (IQR) because its distribution was strongly right-skewed. ^†^ The mean serum albumin value was calculated from measurements obtained during the cycle immediately prior to the survey and the cycle at the time of the survey. Unless otherwise indicated, values are *n* (%) or mean ± SD. * indicates *p* < 0.05. IQR, interquartile range; SD, standard deviation.

**Table 2:** Weekly exercise volume among participants with and without hyperglycemia.

Category	Normoglycemia	Hyperglycemia	Z	*p*
	(*n* = 129)	(*n* = 47)		
	Median (IQR)	Median (IQR)		
Weekly exercise frequency (times/week)	6 (3–7)	0 (0–5)	5.42	<0.001
Exercise duration per session (min/session)	60 (40–90)	0 (0–30)	6.71	<0.001
Weekly exercise volume (kcal/week)	1680 (987–2380)	0 (0–723)	7.29	<0.001

Values are presented as medians (interquartile ranges). Because the Shapiro–Wilk test indicated non-normal distributions (all *p* < 0.001), groups were compared using the Mann–Whitney U test. Z denotes the standardized Mann–Whitney statistic.

**Table 3:** Factor analysis of food-group intakes among participants.

Food Group	Communality	Factor				
		Factor 1 (Korean Side-Dish Pattern)	Factor 2 (Grain–Sugar and Fish Pattern)	Factor 3 (Dairy–Fruit Pattern)	Factor 4 (Nuts/Seeds–Tubers Pattern)	Factor 5 (Legume Pattern)
Oils/fats (g/day)	0.886	0.902	0.157	0.194	0.099	−0.020
Seaweed (g/day)	0.657	0.787	0.001	0.098	−0.133	−0.100
Mushrooms (g/day)	0.689	0.711	−0.168	−0.139	0.362	−0.073
Vegetables (g/day)	0.757	0.652	0.393	0.147	0.286	0.273
Condiments and seasonings (g/day)	0.775	0.596	0.475	0.209	0.199	0.333
Meats (g/day)	0.407	0.500	0.375	0.079	0.056	0.084
Eggs (g/day)	0.495	0.456	0.293	0.416	−0.090	0.141
Grains (g/day)	0.725	0.114	0.839	−0.077	0.010	−0.051
Sugars (g/day)	0.501	0.027	0.635	0.152	0.076	−0.260
Fish and shellfish (g/day)	0.634	0.342	0.508	0.185	0.301	0.366
Milk and dairy products (g/day)	0.681	0.038	−0.009	0.766	0.026	−0.305
Fruits (g/day)	0.544	0.180	0.058	0.673	0.140	0.187
Nuts and seeds (g/day)	0.792	0.129	0.063	0.062	0.869	−0.107
Potatoes and tubers (g/day)	0.488	−0.005	0.276	0.361	0.445	0.288
Legumes (g/day)	0.648	−0.017	−0.166	−0.042	−0.060	0.784
Eigenvalue		3.358	2.136	1.550	1.365	1.269
Variance explained (%)		22.39	14.24	10.33	9.10	8.46
Cumulative variance explained (%)		22.39	36.63	46.96	56.06	64.52
Kaiser–Meyer–Olkin = 0.794		Bartlett’s test χ^2^ = 951.57 (*df* = 105, *p* < 0.001)				

Shaded cells indicate the factor to which each food group was assigned (i.e., the factor with the highest absolute loading ≥ 0.40).

**Table 4:** Associations of dietary pattern, clinical factors, and behavioral factors with hyperglycemia among patients undergoing chemotherapy.

Variable/Exposure	Primary Model		Sensitivity Model (+Confounders)	
Dietary Pattern and Clinical/Behavioral Factors	OR (95% CI)	*p*	OR (95% CI)	*p*
Korean side-dish pattern (Factor 1, per 1 SD increase in the pattern score)	0.30 (0.14–0.65) *	0.002	0.25 (0.11–0.58) *	0.001
Active (vs. inactive)	0.13 (0.04–0.40) *	<0.001	0.08 (0.02–0.30) *	<0.001
Highly active (vs. inactive)	0.06 (0.01–0.31) *	<0.001	0.03 (0.01–0.21) *	<0.001
Serum albumin (per g/dL)	0.17 (0.03–0.89) *	0.036	0.09 (0.01–0.56) *	0.011
Age 60–69 (vs. ≤59)	1.73 (0.43–6.94)	0.442	1.27 (0.30–5.45)	0.746
Age ≥ 70 (vs. ≤59)	3.61 (0.85–15.28)	0.081	3.24 (0.68–15.50)	0.141
Elementary	1.71 (0.28–10.41)	0.560	2.20 (0.33–14.65)	0.416
Middle school	1.06 (0.17–6.72)	0.952	1.14 (0.17–7.73)	0.897
High school	0.84 (0.15–4.62)	0.843	1.11 (0.19–6.48)	0.908
Female (vs. male)	—	—	0.37 (0.12–1.12)	0.079
BMI (per kg/m^2^)	—	—	1.11 (0.96–1.28)	0.141
GI cancer (vs. other)	—	—	3.62 (1.10–11.85) *	0.034
Stage IV (vs. I–III)	—	—	0.56 (0.17–1.82)	0.333
Chemotherapy cycles (per cycle)	—	—	1.02 (0.95–1.10)	0.582

Odds ratios for the primary model are adjusted for age, education, physical activity level, and serum albumin. The sensitivity model additionally adjusts for sex, BMI, cancer diagnosis (gastrointestinal vs. other), disease stage, and number of chemotherapy cycles; these confounders were prespecified on clinical grounds rather than through univariate screening. The dietary-pattern score was standardized, and its OR represents the change in odds per 1 SD increase in the pattern score. — indicates a variable not included in this model. OR, odds ratio; CI, confidence interval. * *p* < 0.05.

**Table 5:** Associations between selected food-group intakes and hyperglycemia in patients undergoing chemotherapy, estimated using a multivariable- and energy-adjusted food-group model.

Food Group (per 1 SD)	OR (95% CI)	*p*	FDR *q*
Oils/fats	0.31 (0.16–0.57) *	<0.001	0.002
Vegetables	0.30 (0.15–0.57) *	<0.001	0.002
Eggs	0.34 (0.16–0.71) *	0.004	0.015
Grains	1.90 (1.19–3.02) *	0.007	0.020
Seaweed	0.32 (0.14–0.75) *	0.008	0.020
Mushrooms	0.64 (0.34–1.20)	0.165	0.331
Seeds/nuts	0.80 (0.49–1.30)	0.366	0.585
Fish/shellfish	0.80 (0.49–1.32)	0.390	0.585
Milk	0.84 (0.51–1.40)	0.513	0.667
Sugars	1.13 (0.75–1.69)	0.556	0.667
Meats	0.91 (0.57–1.44)	0.685	0.748
Condiments/seasonings	1.04 (0.61–1.77)	0.877	0.877

Odds ratios are adjusted for age, education, physical activity level, and serum albumin. Energy-adjusted using the residual method, continuous dietary exposures were standardized (OR per 1 SD). Although this table presents selected food groups, the FDR correction was applied across all 15 food-group tests. FDR, false discovery rate; OR, odds ratio; CI, confidence interval; Benjamini–Hochberg correction applied. * *p* < 0.05.

**Table 6:** Exploratory associations between selected nutrient intakes and hyperglycemia in patients undergoing chemotherapy, estimated using a nutrient model with multivariable adjustment and energy adjustment.

Nutrient (per 1 SD)	OR (95% CI)	*p*	FDR *q*
Vitamin E	0.48 (0.27–0.86)	0.013	0.154
Cholesterol	0.50 (0.28–0.89)	0.020	0.154
Folate	0.43 (0.21–0.88)	0.021	0.154
Dietary fiber	0.43 (0.20–0.92)	0.029	0.159
Magnesium	0.59 (0.35–1.00)	0.050	0.216
Ash	0.61 (0.36–1.02)	0.060	0.216
Histidine	0.66 (0.41–1.07)	0.089	0.216
Lysine	0.64 (0.39–1.07)	0.091	0.216
Valine	0.66 (0.41–1.08)	0.097	0.216
Iron	0.61 (0.34–1.10)	0.098	0.216
Leucine	0.67 (0.41–1.09)	0.108	0.216
Total fatty acids	0.65 (0.38–1.12)	0.123	0.226
Phenylalanine	0.69 (0.43–1.12)	0.136	0.230
Tyrosine	0.70 (0.43–1.14)	0.153	0.240
Copper	0.72 (0.44–1.20)	0.210	0.308
Glycine	0.75 (0.46–1.22)	0.245	0.324
Alanine	0.76 (0.47–1.22)	0.258	0.324
Vitamin B6	0.74 (0.44–1.25)	0.265	0.324
Cysteine	0.80 (0.50–1.29)	0.364	0.422
Pantothenic acid	0.82 (0.51–1.32)	0.406	0.447
Fluorine	1.14 (0.75–1.73)	0.544	0.570
Taurine	0.89 (0.51–1.54)	0.680	0.680

Odds ratios were adjusted for age, education, physical activity level, and serum albumin. Continuous dietary exposures were energy-adjusted using the residual method and standardized, with ORs expressed per 1 SD. Although this table presents selected nutrients, the FDR correction was applied across all nutrients tested. All nutrient associations are exploratory, and none survived FDR correction (all *q* > 0.05). FDR denotes false discovery rate; the Benjamini–Hochberg correction was applied. OR and CI denote odds ratio and confidence interval, respectively.

## Data Availability

The data presented in this study are available on request from the corresponding author due to privacy and ethical restrictions concerning the study participants.

## Supplementary Materials

The following supporting information can be downloaded at: https://www.mdpi.com/article/10.3390/nu18193249/s1, Table S1: Descriptive statistics for the continuous dietary exposures; Table S2: Extended sensitivity analysis including confounders (clinical factors, other medications, and cumulative dexamethasone dose).

## Abbreviations

The following abbreviations are used in this manuscript:

BMI	Body mass index
CI	Confidence interval
EHR	Electronic health records
FDR	False discovery rate
FFQ	Food frequency questionnaire
FPG	Fasting plasma glucose
GI	Gastrointestinal
HbA1c	Glycated hemoglobin
IQR	Interquartile range
IRB	Institutional Review Board
KNHANES	Korea National Health and Nutrition Examination Survey
OR	Odds ratio
PCA	Principal component analysis
RPG	Random plasma glucose
SD	Standard deviation
STROBE-nut	Strengthening the Reporting of Observational Studies in Epidemiology—Nutritional Epidemiology
