# Supplementary material for: Associations of Dietary Patterns, Daily Nutrient Intakes, and Food-Group Intakes with Hyperglycemia in Patients Undergoing Chemotherapy
Source: Nutrients. 2026 Oct 1;18(19):3249. doi: 10.3390/nu18193249 (PMC13666821; doi:10.3390/nu18193249)
Supplement: Supplementary file 1 [file nutrients-18-03249-s001.zip › nutrients-4529832-supplementary.pdf]

Supplementary Table S1. Descriptive statistics for the continuous dietary exposures.

Dietary exposure	Mean ± SD	Median (IQR)
Food groups (g/day)		
Oils/fats	16.3 ± 11.0	14.0 (8.4–21.7)
Seaweed	11.6 ± 13.5	9.6 (1.3–14.6)
Mushrooms	4.9 ± 5.5	3.2 (1.1–6.1)
Vegetables	414.6 ± 229.6	362.1 (276.9–518.6)
Condiments and seasonings	46.7 ± 28.3	41.4 (28.9–56.2)
Meats	219.1 ± 151.9	189.2 (118.4–268.2)
Eggs	60.7 ± 48.7	50.6 (30.8–83.0)
Grains	285.9 ± 124.8	260.9 (190.7–363.9)
Sugars	6.9 ± 7.7	4.8 (2.2–8.3)
Fish and shellfish	68.9 ± 51.9	57.1 (30.9–89.2)
Milk and dairy products	93.4 ± 111.3	80.0 (0.5–151.4)
Fruits	299.8 ± 309.0	228.7 (104.1–370.6)
Nuts and seeds	18.2 ± 38.0	1.4 (0.4–19.9)
Potatoes and tubers	75.8 ± 67.5	60.3 (25.1–104.9)
Legumes	178.4 ± 179.0	124.8 (65.7–244.3)
Nutrients (per day)		
Vitamin E (mg)	24.7 ± 13.1	22.4 (15.4–31.7)
Cholesterol (mg)	584.1 ± 356.0	490.8 (353.9–757.2)
Folate (µg)	863.6 ± 435.7	768.0 (566.1–1061.4)
Dietary fiber (g)	37.4 ± 18.3	33.9 (25.9–45.2)
Magnesium (mg)	149.6 ± 105.6	130.9 (79.7–189.2)
Ash (g)	31.8 ± 14.7	29.9 (20.5–39.6)
Histidine (mg)	3210.6 ± 1572.1	2960.9 (2229.2–3761.6)
Lysine (mg)	5134.4 ± 2808.8	4577.2 (3177.2–6217.3)
Valine (mg)	5040.2 ± 2761.7	4512.8 (3368.7–6176.1)
Iron (mg)	26.5 ± 11.8	23.7 (18.6–33.4)
Leucine (mg)	7228.4 ± 3647.5	6480.0 (4820.0–8588.7)
Total fatty acids (g)	44.8 ± 28.1	41.8 (25.1–57.5)
Phenylalanine (mg)	4236.5 ± 2251.8	3752.4 (2849.7–5080.9)
Tyrosine (mg)	3103.2 ± 1555.6	2762.1 (2098.6–3763.3)
Copper (mg)	2.0 ± 1.0	1.8 (1.4–2.3)
Glycine (mg)	3814.4 ± 1923.4	3465.2 (2495.6–4679.9)
Alanine (mg)	5086.1 ± 2387.0	4473.3 (3377.4–6150.5)
Vitamin B6 (mg)	2.5 ± 1.1	2.3 (1.8–3.2)
Cysteine (mg)	984.2 ± 475.3	874.8 (668.8–1192.7)
Pantothenic acid (mg)	7.5 ± 2.9	7.2 (5.6–8.9)
Fluorine (µg)	26.4 ± 18.4	23.6 (11.2–36.0)
Taurine (mg)	22.5 ± 51.0	0.0 (0.0–11.3)

Values are presented as mean ± standard deviation (SD) and median (interquartile range [IQR]) for the absolute (unadjusted) daily intakes (N = 176). In the logistic regression analyses, these exposures were energy-adjusted by the residual method and expressed per 1 SD. Amino acids are expressed in mg/day. As a sensitivity check for the five food groups that remained significant after FDR correction, energy-adjusted intakes were categorized into quartiles and

entered as ordinal variables; all five exposures showed significant monotonic trends (all P for trend < 0.05). This analysis assesses monotonic dose–response patterns but does not constitute a formal test of linearity in the logit.

Supplementary Table S2. Extended sensitivity analysis including confounders (clinical factors, other medications, and cumulative dexamethasone dose).

Variable	Standard logistic		Firth penalized	
	OR (95% CI)	*p*	OR (95% CI)	*p*
Korean side-dish pattern (Factor 1, per 1 SD increase in the pattern score)	0.14 (0.04–0.57)*	0.006	0.27 (0.10–0.72)*	0.009
Age 60–69 (vs ≤59)	1.03 (0.13–7.92)	0.978	1.07 (0.20–5.78)	0.941
Age ≥70 (vs ≤59)	2.40 (0.27–21.24)	0.432	1.88 (0.31–11.34)	0.490
Elementary	3.70 (0.19–72.19)	0.388	2.28 (0.22–23.41)	0.489
Middle school	0.48 (0.02–10.03)	0.640	0.54 (0.05–5.99)	0.618
High school	0.72 (0.05–10.73)	0.810	0.73 (0.09–5.98)	0.773
Active (vs inactive)	0.02 (0.00–0.17)*	<0.001	0.06 (0.01–0.29)*	<0.001
Highly active (vs inactive)	0.00 (0.00–0.07)*	<0.001	0.02 (0.00–0.17)*	<0.001
Serum albumin (per g/dL)	0.01 (0.00–0.23)*	0.003	0.05 (0.01–0.41)*	0.006
Female (vs male)	0.06 (0.01–0.40)*	0.003	0.15 (0.04–0.59)*	0.007
BMI (per kg/m^2)	1.12 (0.87–1.43)	0.376	1.09 (0.90–1.31)	0.390
GI cancer (vs other)	21.39 (2.78–164.78)*	0.003	8.67 (1.82–41.28)*	0.007
Stage IV (vs I–III)	0.85 (0.15–4.93)	0.860	0.89 (0.23–3.52)	0.874
Chemotherapy cycles (per cycle)	0.92 (0.81–1.06)	0.253	0.95 (0.85–1.06)	0.350
Antihypertensive med. (yes vs no)	1.20 (0.23–6.14)	0.826	1.18 (0.31–4.55)	0.807
Immunotherapy (yes vs no)	68.50 (1.34–3514.09)*	0.035	21.88 (0.95–506.04)	0.054
Cumulative dexamethasone (log mg)	1.44 (0.77–2.68)	0.255	1.28 (0.76–2.17)	0.356

*All variables entered simultaneously; the model was adjusted for the prespecified covariates (age, education, physical-activity level, and serum albumin) together with the reviewer-requested confounders. The dietary-pattern score was derived from absolute (unadjusted) food-group intakes and standardized; the OR for the Korean side-dish pattern represents the change in odds per 1 SD increase in the pattern score. Both standard maximum-likelihood and Firth penalized logistic regression are shown. The core associations (Korean side-dish pattern, physical activity, serum albumin, and female sex) remained significant under both methods, and cumulative dexamethasone was non-significant under both (standard OR 1.44, p =.255; Firth OR 1.28, p =.356). Firth's penalization substantially stabilized the estimates for sparse variables (e.g., immunotherapy: standard OR 68.50, 95% CI 1.34–3514.09 vs Firth OR 21.88, 95% CI 0.95–506.04), confirming that the conclusions are robust to the low events-per-variable ratio; this fully adjusted model is nonetheless presented only as a sensitivity analysis. OR, odds ratio; CI, confidence interval. * $p < 0.05$.*
